# Regulation of TIMP-1 in Human Placenta and Fetal Membranes by lipopolysaccharide and demethylating agent 5-aza-2'-deoxycytidine

**DOI:** 10.1186/s12958-015-0132-y

**Published:** 2015-12-21

**Authors:** Zoë L. Vincent, Murray D. Mitchell, Anna P. Ponnampalam

**Affiliations:** Liggins Institute, University of Auckland, Private Bag 92019, Auckland, 1142 New Zealand; Gravida: National Centre for Growth and Development, Palmerston North, New Zealand; University of Queensland Centre for Clinical Research, Brisbane, Australia; The Heart Foundation, Auckland, New Zealand

**Keywords:** TIMP1, Placenta, Labour, Epigenetics, Infection

## Abstract

**Background:**

An appropriate transcriptional profile in the placenta and fetal membranes is required for successful pregnancy; any variations may lead to inappropriate timing of birth. Epigenetic regulation through reversible modification of chromatin has emerged as a fundamental mechanism for the control of gene expression in a range of biological systems and can be modified by pharmacological intervention, thus providing novel therapeutic avenues. TIMP-1 is an endogenous inhibitor of MMPs, and hence is intimately involved in maintaining the integrity of the fetal membranes until labor.

**Objective and Methods:**

To determine if TIMP-1 is regulated by DNA methylation in gestational tissues we employed an *in vitro* model in which gestational tissue explants were treated with demethylating agent 5-aza-2'-deoxycytidine (AZA) and lipopolysaccharide (LPS).

**Results:**

Quantitative Real-Time PCR (qRT-PCR) revealed that TIMP-1 transcription was significantly increased by combined treatment of AZA and LPS, but not LPS alone, in villous, amnion and choriodecidua explants after 24 and 48 hrs, whilst western blotting showed protein production was stimulated after 24 hrs only. Upon interrogation of the TIMP-1 promoter using Sequenom EpiTyper MassARRAY, we discovered sex-specific differential methylation, in part explained by x-linked methylation in females. Increased TIMP-1 in the presence of LPS was potentiated by AZA treatment, signifying that a change in chromatin structure, but not in DNA methylation at the promoter region, is required for transcriptional activators to access the promoter region of TIMP-1.

**Conclusions:**

Collectively, these observations support a potential role for pharmacological agents that modify chromatin structure to be utilized in the therapeutic targeting of TIMP-1 to prevent premature rupture of the fetal membranes in an infectious setting.

## Background

The end of human gestation is signified by the physiological hallmarks of uterine contractions and rupture of the fetal membranes (amnion and chorion) [1]. These two processes are essential for the advancement of labor, and inappropriate timing of these processes may lead to premature or post-term birth, both of which have adverse consequences for the neonate [1, 2].

Matrix metalloproteinase (MMP) mediated extracellular matrix (ECM) degradation plays a key role in normal growth and remodelling of fetal membranes throughout gestation and at term, and are critically involved in the weakening and subsequent rupture of the fetal membranes at the time of labor [3]. Locally produced tissue inhibitors of metalloproteinases (TIMPs) function as critical modulators of both normal and pathological tissue remodelling. As TIMPs bind MMPs in a 1:1 stoichiometric fashion, thus an imbalanced MMP:TIMP ratio can result in tissue degradation.

TIMP-1 levels decrease in the amniotic fluid with advancing gestation, concomitant with a marked increase in MMP-9 prior to the onset of labor [4–8]. Increased MMPs, particularly MMP-9, in fetal membrane rupture at both preterm and term labor is well documented [6, 7, 9–13] and the MMP-9:TIMP-1 ratio in fetal membranes at term gestation correlates with their tensile strength [14]. Furthermore, this altered MMP-9:TIMP-1 ratio is exacerbated in the presence of infection [7, 15–19].

Preterm rupture of the fetal membranes (PROM) occurs in as many as 15 % of all pregnancies and is associated with 25-50 % of preterm births [20, 21]. The etiology of preterm labor is complex and multifactorial, however intrauterine infection caused by bacteria is the leading identifiable cause [1, 22]. Lipopolysaccharide (LPS), an endotoxin on the outer surface of gram negative bacteria, has been implicated in the mechanism responsible for PROM and preterm delivery [23, 24].

Epigenetic regulation through the reversible modification of chromatin has emerged as a fundamental mechanism for the control of gene expression in a range of biological systems. DNA methylation is the covalent modification of post-replicative DNA by the addition of a methyl group to the cytosine ring to form methyl cytosine, usually in the context of CpG dinucleotides. Discrete CpG rich regions, approximately 1 kb in size, are known as CpG islands (CGIs) and occur in more than half of the genes in the vertebrate genome [25]. CGIs are usually unmethylated, and the extent of methylation in promoter regions is generally inversely correlated with gene activity [26, 27]. Epigenetic modifications are subject to both developmental and environmental regulation, are reversible and can potentially be modified, thereby making them an attractive target for therapeutic interventions.

DNA methylation is essential for normal development of extra-embryonic tissues, particularly the invasive behavior of trophoblast cells [28]. The placenta is unique in that it is hypomethylated compared to somatic tissues [29, 30], and DNA methylation is positively correlated with gestational age [31, 32]. This suggests that there are consistent and large scale changes to DNA methylation in the placenta throughout pregnancy, contributing to differential global gene expression across gestation [33] and at term prior to, and following, spontaneous labor and delivery [34, 35].

Silencing of TIMP-1 by promoter methylation is a feature of cancer cells, and treatment with demethylating agent AZA results in the induction of TIMP-1 in a number of cancer cell lines [36–40] as well as endometrial stromal cells [38].

Given that aberrant placental methylation has been associated with adverse pregnancy outcomes [39–41], and TIMP-1 is regulated by DNA methylation in other tissues; we hypothesize that TIMP-1 is regulated by DNA methylation in the placenta and fetal membranes, and that this regulation is altered in the presence of infection.

## Methods

### Tissue Collection and explant system

Gestational tissues (villous placenta and fetal membranes) were collected from women at term with uncomplicated, singleton pregnancies (38-40 weeks gestation) following elective caesarean section (pre-labor, CS) and after vaginal delivery from women with spontaneous onset of labor (post-labor and delivery, SVD). Indications for cesarean section were breech presentation or previous cesarean section delivery. Women were excluded from the study if they had a twin pregnancy, became pregnant using in vitro fertilization, suffered any pregnancy complications including preeclampsia or gestational diabetes, were a current smoker, or smoked up until, or during any part of their pregnancy. Ethical approval for the study was obtained from NorthernX Regional Ethics Committee (NTX/10/07/062/AM03) and tissues were collected following written informed consent. All the participants have given consent to publish the data. The fetal membranes were separated into amnion and chorion (with the immediately adjacent decidua attached, herein referred to as choriodecidua), and all tissues were rinsed in PBS to remove maternal blood. Tissues from both CS and SVD deliveries were snap frozen and stored at -80 C until further analysis. Tissues from CS deliveries were cultured using a previously published *in vitro* tissue explant system [42–44] with modifications as follows.

Samples of villous tissue were taken randomly across the placenta from mid-sections of cotyledons (halfway between the maternal and fetal sides). Large vessels were removed using blunt dissection leaving only villous tissue, which was further dissected into 20 mg pieces. Fetal membranes were separated into amnion and choriodecidua and 6 mm tissue discs were excised using a sterile cork borer. Villous, amnion and choriodecidua explants were plated separately (six pieces per well) and equilibrated in DMEM/F12 containing L-Glutamate (Life Technologies, Carlsbad, CA, USA) with 10 % FBS (Life Technologies) and 1 % Penstrep solution (final concentrations 100 U/ml Penicillin and 100 μg streptomysin; Life Technologies) in a humidified atmosphere of 5 % CO_2_ and 8 % O_2_ for 24 hrs.

After equilibration, tissues were washed and media were replaced with DMEM/F12 supplemented with 0.1 % bovine gamma globulin (Sigma–Aldrich, St. Louis, MO, USA) containing 5 μM AZA (Sigma–Aldrich) or DMSO as the control (Sigma–Aldrich). Media for all treatments contained 0.05 % DMSO. The dose and length of AZA treatment was based on previous publications [45]. Following 48 hrs culture, tissues were extensively washed in sterile PBS and tissues were further incubated in the presence or absence of 5 μg/ml LPS (E.coli, Sigma–Aldrich). Tissues were cultured with LPS to determine if an inflammatory response induced changes in TIMP-1 expression and/or DNA methylation. Control tissues were cultured in DMSO only for the duration of the culture period, with exception of the initial 24 hrs equilibration period. Culture was terminated at 24 hrs and 48 hrs post LPS treatment, tissues were snap frozen and conditioned media reserved. Tissues and media samples were stored at -80C and -20C respectively.

### Glucose uptake by tissue explants

Tissue viability was assessed by glucose uptake in conditioned media from explant experiments (Reti, Lappas, Huppertz, et al., 2007). Glucose uptake was measured by enzymatic colourimetric assay (Roche, Mannheim, Germany) on a Hitachi 902 autoanalyser (Hitachi High Technologies Corporation, Tokyo, Japan). Data were normalised to wet tissue weight and minutes (Table 1).

**Table 1:** Glucose uptake by gestational tissue explants

	24 hrs				48 hrs			
	Control	AZA	LPS	AZA + LPS	Control	AZA	LPS	AZA + LPS
Placenta	0.80 ± 0.13	0.92 ± 0.09	0.79 ± 0.08	0.86 ± 0.06	0.43 ± 0.04	0.63 ± 0.02	0.49 ± 0.05	0.48 ± 0.06
Amnion	0.98 ± 0.02	0.92 ± 0.02	0.86 ± 0.03	0.91 ± 0.02	0.84 ± 0.02	0.85 ± 0.01	0.76 ± 0.02	0.73 ± 0.01
Choriodecidua	0.43 ± 0.03	0.47 ± 0.07	0.41 ± 0.06	0.40 ± 0.06	0.38 ± 0.05	0.41 ± 0.04	0.39 ± 0.03	0.45 ± 0.04

Glucose uptake by placenta, amnion and choriodecidua explants was measured in conditioned culture media by enzymatic colourimetric assay.

Data are presented as glucose uptake μmol/mg/min (mean ± SEM; n = 8)

### RNA Extraction and Real time PCR

Total RNA was isolated from tissues using Trizol® (Life Technologies) according to manufacturer’s instructions. RNA concentrations were quantified using a NanoDrop ND-1000 spectrophotometer (NanoDrop, Thermo Scientific, USA). Reverse transcription and cDNA synthesis was performed using Transcriptor First Strand cDNA Synthesis Kit (Roche Applied Sciences, Penzberg, Germany) according to manufacturer’s instructions using 1 μg of total RNA for each preparation. The resulting cDNA was stored at -20C until required.

TIMP-1 expression was analysed by Quantitative Real-Time PCR (qRT-PCR) using the LightCycler 480, LightCycler 480 SYBR Green Master Mix (Roche Applied Sciences). Gene specific primers used for qRT-PCR are as follows. TIMP-1: sense 5’-TCTGGCATCCTGTTGTTGCT-3’; antisense 5’-CGCTGGTATAAGGTGGTCTGG-3’. RPLPO: sense 5’-AGAAACTGCTGCCTCATATCCG-3’; antisense 5’-CCCCTGGAGATTTTAGTGGTGA-3’. RPL13a: sense 5’-GCCCTACGACAAGAAAAAGCG-3’; antisense 5’-TACTTCCAGCCAACCTCGTGA-3’ (Integrated DNA Technologies, Custom Science, Auckland, New Zealand). Primer specificity was confirmed by the Massey Genome Service at Massey University, Palmerston North, New Zealand. RPLPO and RPL13a were used as endogenous controls to normalise gene expression. The average transcript quantity in tissues collected from CS and SVD deliveries and in treated tissue explants was calculated using the relative standard curve method and Delta-delta CT method respectively, normalised to the geometric mean of the endogenous controls.

### Western blotting

Western blotting was performed on whole cell lysates using rabbit monoclonal anti-TIMP-1 antibody (ab109125, Abcam, Cambridge, UK). Samples were separated by weight by SDS-PAGE using 4-12 % Bis-Tris gels (Life Technologies), and transferred onto PVDF membrane. Following pre-incubation with a blocking solution, membranes were incubated in primary antibody overnight. The membranes were then extensively washed, incubated in HRP-conjugated secondary antibody (A5045, Sigma Aldrich) and were visualized using Pierce SuperSignal West Dura Extended Duration Substrate (Thermo Scientific, Illinois, USA). Relative protein levels were obtained using densitometric quantification (Quantity One software; Bio-Rad Laboratories, Hercules, California, USA) and were normalized to beta actin.

### DNA Extraction and Methylation Analysis

Genomic DNA was extracted from tissues using Qiagen QiaAMP DNA extraction kit (QIAGEN, Hilden, Germany) as per manufacturers’ instructions. DNA concentrations were quantified using a NanoDrop ND-1000 spectrophotometer.

**Fig. 1:**
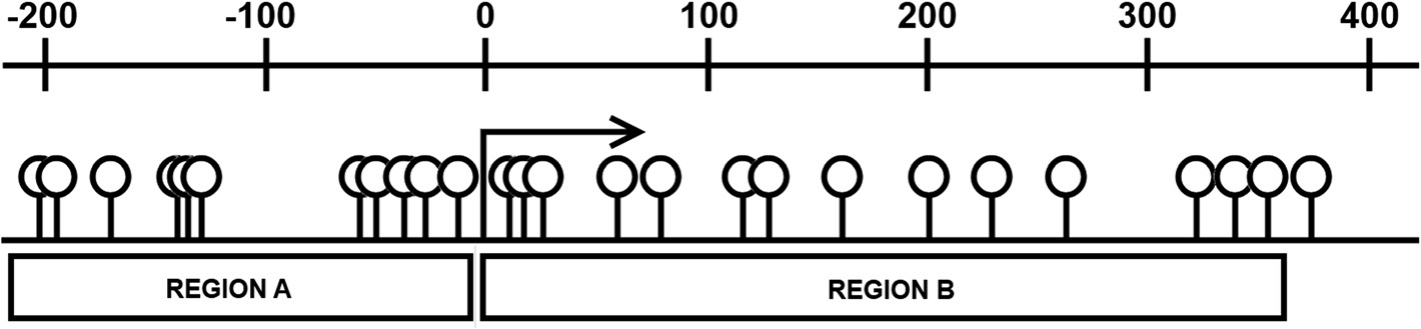
Schematic of the TIMP-1 gene promoter and locations of primers used for methylation analysis. Region A is upstream of the transcription start site (-275/-1) and covers 11 CpGs, Region B (+1/+279) covers the TIMP-1 promoter and first exon and contains 14 CpGs. The numbered line represents the distance in base pairs from the transcription start site, shown by the number 0. CpGs are represented by open circles

Methylation analysis using the Sequenom™ EpiTyper® MassARRAY platform was undertaken by the Australian Genome Research Facility (AGRF; www.agrf.org.au). Sequenom primers specific to two regions of the TIMP-1 gene were designed in-house by AGRF. Region A is upstream from the transcription start site (-275/-1) covering 11 CpGs and Region B includes the TIMP-1 promoter and first exon, covering 14 CpGs (Fig. 1). Methylation analysis was carried out on bisulphite converted DNA, using 200 ng for each preparation. Methylation levels for each CpG unit are expressed as a percentage which is calculated from the ratio mass signals between methylated and non-methylated DNA in each sample.

### Statistical Analysis

Data are presented as means ± SEM and statistical comparisons between groups were performed using non-parametric Kruskal Wallis ANOVA (1-way analysis of variance) with Dunn’s post-hoc test using GraphPad Prism (GraphPad Software, La Jolla California USA, www.graphpad.com). An alpha value of less than 0.05 was considered significant.

## Results

### Expression of TIMP-1 in Gestational Tissues in relation to labor

**Fig. 2:**
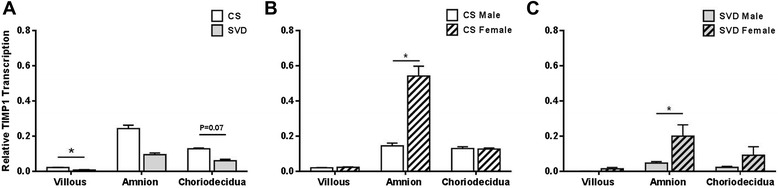
TIMP-1 mRNA Expression in Term Villous Placenta, Amnion and Choriodecidua measured by qRT-PCR. **a** Tissues collected prior to the onset of labor from elective Caesarean sections deliveries (CS, n = 14) are shown by the white bars. Tissues collected following the spontaneous onset of labor from vaginal deliveries (SVD, n = 10) are shown by grey bars. TIMP-1 transcription was further analysed by sex of the fetus in **b** CS deliveries (Male n = 8, Female n = 6) and **c** SVD (Male n = 5 and Female n = 5). Data are presented as mean ± SEM * P ≤ 0.05. All results were normalised to the expression of endogenous controls (RPLPO and RPL13a)

TIMP-1 mRNA levels were significantly higher in villous tissues collected from elective caesarean section deliveries, prior to the onset of labor (CS) compared to tissues collected following spontaneous vaginal delivery (SVD). This trend was observed also in choriodecidua, but not in amnion tissues (Fig. 2a). Tissues from both CS and SVD had significantly higher TIMP-1 mRNA in female amnion compared to male amnion (Fig. 2b and c).

### Effect of AZA and LPS treatments on TIMP-1 transcription and protein production

**Fig. 3:**
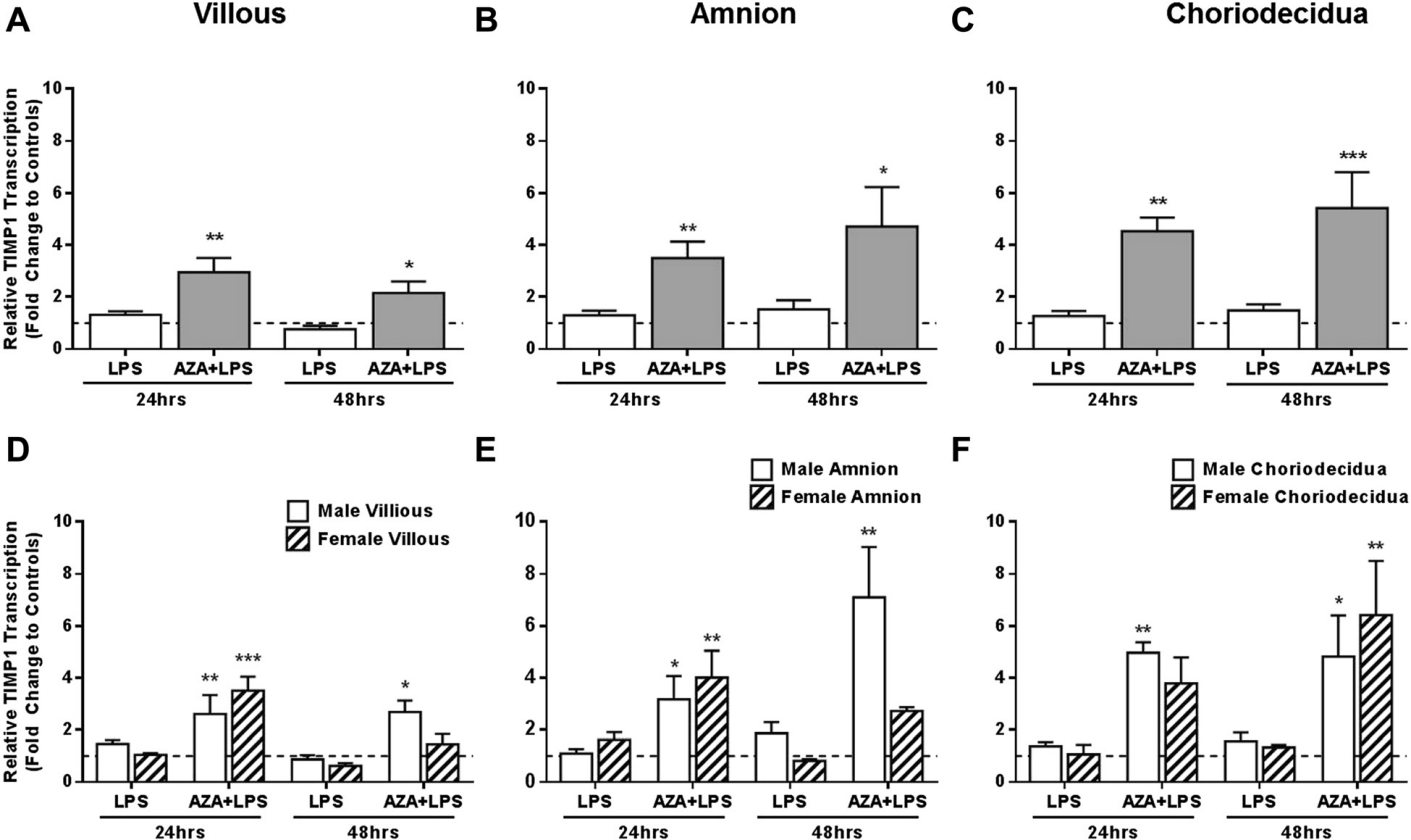
TIMP-1 mRNA expression as fold change compared to controls in gestational tissues measured using qRT-PCR. Tissues were cultured in the presence of lipopolysaccharide (LPS, 5 μg/ml) with/without prior treatment with 5-aza-deoxycytidine (AZA, 5 μM; n = 8). **a** Villous placenta **b** Amnion and **c** Choriodecidua, and further analysed by the sex of the fetus in **d** Villous placenta **e** Amnion and **f** Choriodecidua. Tissues from pregnancies with male fetuses (n = 5) are shown by white bars and those from pregnancies with female fetuses (n = 3) are shown by cross-hatched bars. Data are calculated as fold change compared to time matched DMSO treated control, represented by the dotted line, and presented as mean ± SEM * P ≤ 0.05, ** P ≤ 0.01, *** P ≤ 0.001. All results were normalised to the expression of endogenous controls (RPLPO and RPL13a)

Placenta, amnion and choriodecidua tissues pre-treated with AZA and subsequently treated with LPS for both 24 hrs and 48 hrs significantly increased TIMP-1 mRNA expression compared to controls (Fig. 3). LPS treatment alone, without pre-treatment of AZA, had no effect on TIMP-1 transcription. Further scrutiny of the data revealed a greater stimulation of TIMP-1 transcription in male villous and amnion that were pre-treated with AZA compared to female tissues in 48 hour treated tissues (Fig. 3d and e).

**Fig. 4:**
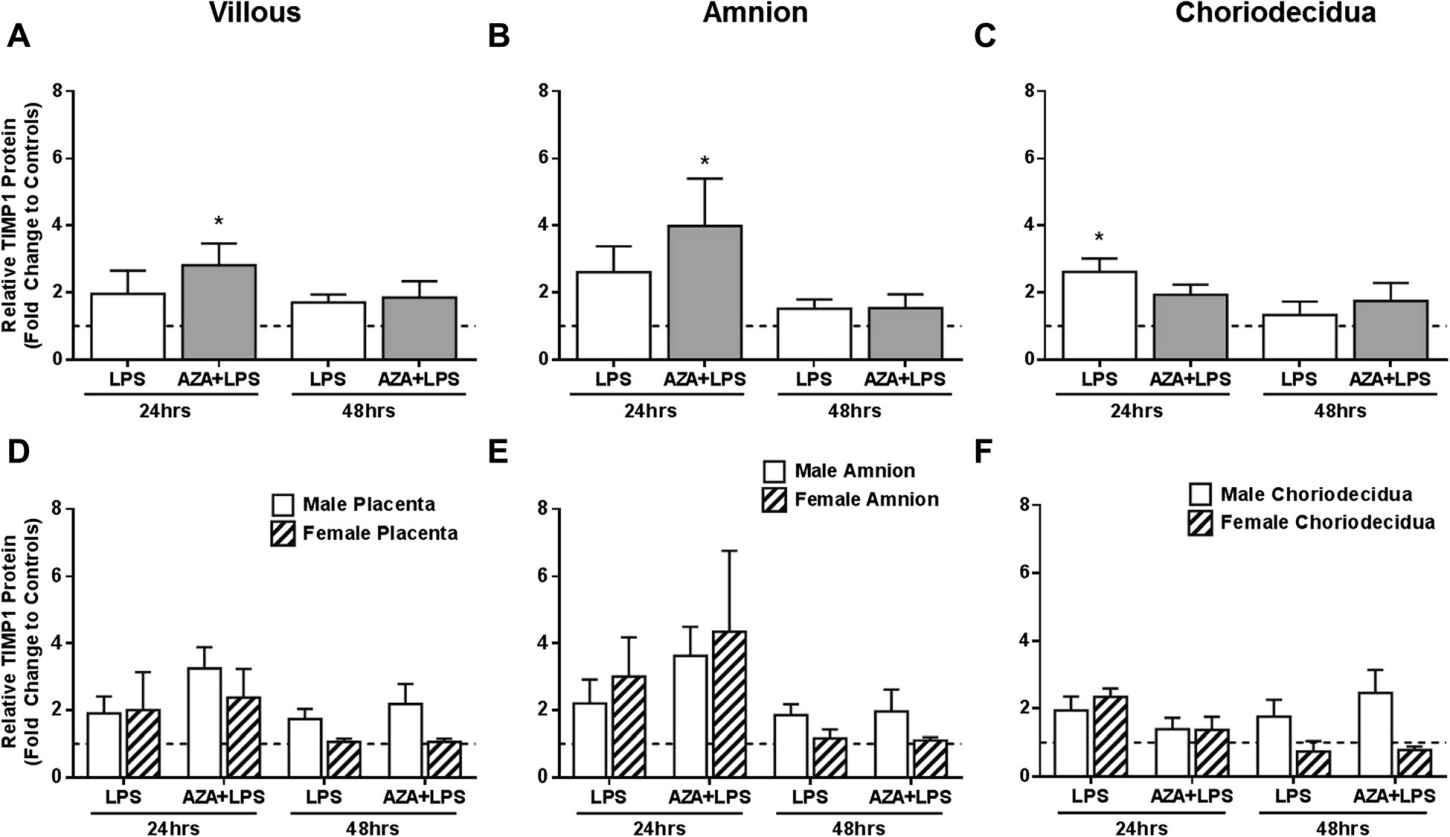
TIMP-1 protein measured by western blotting in treated explant samples shown as fold change compared to controls. Tissues were cultured in the presence of lipopolysaccharide (LPS, 5 μg/ml) with/without prior treatment with 5-aza-deoxycytidine (AZA, 5 μM; n = 8). **a** Villous placenta **b** Amnion and **c** Choriodecidua and further analysed by the sex of the fetus in **d** Villous placenta **e** Amnion and **f** Choriodecidua. Tissues from pregnancies with male fetuses (n = 5) are shown by white bars and those from pregnancies with female fetuses (n = 3) are shown by cross-hatched bars. Data are calculated as fold change compared to time matched DMSO treated control, represented by the dotted line, and presented as mean ± SEM * P ≤ 0.05. All results were normalised to beta actin optical density

Villous and amnion explants pre-treated with AZA and subsequently incubated in the presence of LPS for 24 hrs had significantly higher TIMP-1 protein production compared to controls (Fig. 4a and b). As with the mRNA, LPS treatment alone had little effect on TIMP-1 protein production in villous (Fig. 4a) or amnion explants (Fig. 4b) however did significantly increase TIMP-1 protein in choriodecidual explants following 24 hrs incubation (Fig. 4c). No changes in TIMP-1 protein production were observed in tissues cultured with LPS for 48 hrs (Fig. 4).

Similar to the mRNA data, there appears to be a sex-specific response of TIMP-1 protein production to the treatments, particularly in male choriodecidua treated for 48 hrs (Fig. 4f). Although notable, high variations among sample groups prevent statistical significance.

### Methylation analysis of TIMP-1

**Fig. 5:**
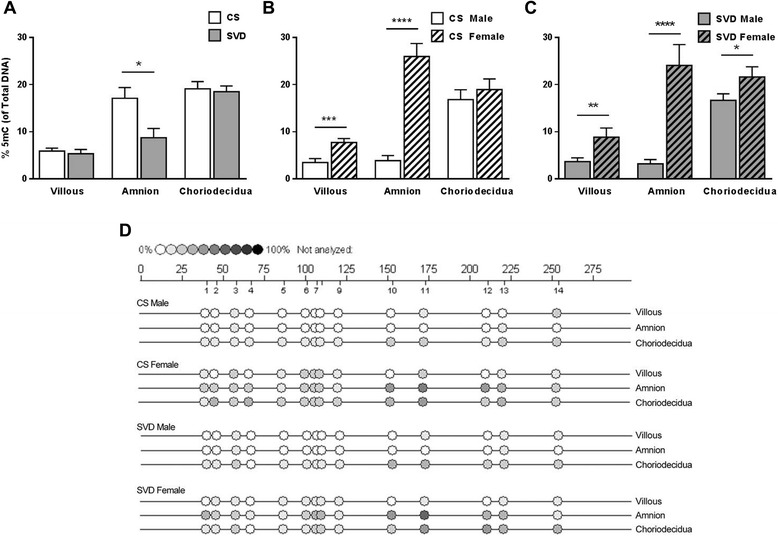
Percentage methylated CpGs in the TIMP-1 Promoter in term villous placenta, amnion and choriodecidua collected prior to, or post labor and delivery. **a** Tissues collected prior to the onset of labor from elective Caesarean sections deliveries (CS, n = 14) are shown by the white boxes. Tissues collected following the spontaneous onset of labor from vaginal deliveries (SVD, n = 10) are shown by grey boxes. TIMP-1 methylation further analysed by sex of the fetus in **b** CS deliveries (Male n = 8, Female n = 6) and **c** SVD (Male n = 5 and Female n = 5). Tissues from pregnancies with male fetuses are shown by white or grey bars and those from pregnancies with female fetuses are shown by cross-hatched bars. **d** Representative epigrams show the % of 5-mC for each tissue at each CpG site within the amplicon for Region B. Percentage methylation calculated from the ratio of mass signals between methylated and non-methylated DNA in each sample. * P ≤ 0.05, ** P ≤ 0.01, *** P ≤ 0.001, **** P ≤ 0.0001. Data are presented as mean %5-mC + SEM

Methylation within the TIMP-1 promoter was significantly higher in amnion from CS deliveries compared to SVD (17 % and 8 % respectively) (Fig. 5a). Villous and placenta from pregnancies with a male fetus were hypomethylated compared to tissues from pregnancies with female fetuses in both CS and SVD samples (CS villous male 3.5 %, female 7.8 %; CS amnion male 3.9 %, female 25.9 %; SVD villous male 3.7 %, female 8.9 %; SVD amnion male 3.2 %, female 24 %). Choriodecidua from pregnancies with male fetuses were hypomethylated compared to choriodecidua from pregnancies with female fetuses in SVD alone (16 % and 22 %, respectively) (Fig. 5b and c). This was evident in both regions of the TIMP-1 promoter analysed (only data from region B is shown). The percentage methylation of each CpG is shown in the representative epigram for each tissue (Fig. 5d).

**Fig. 6:**
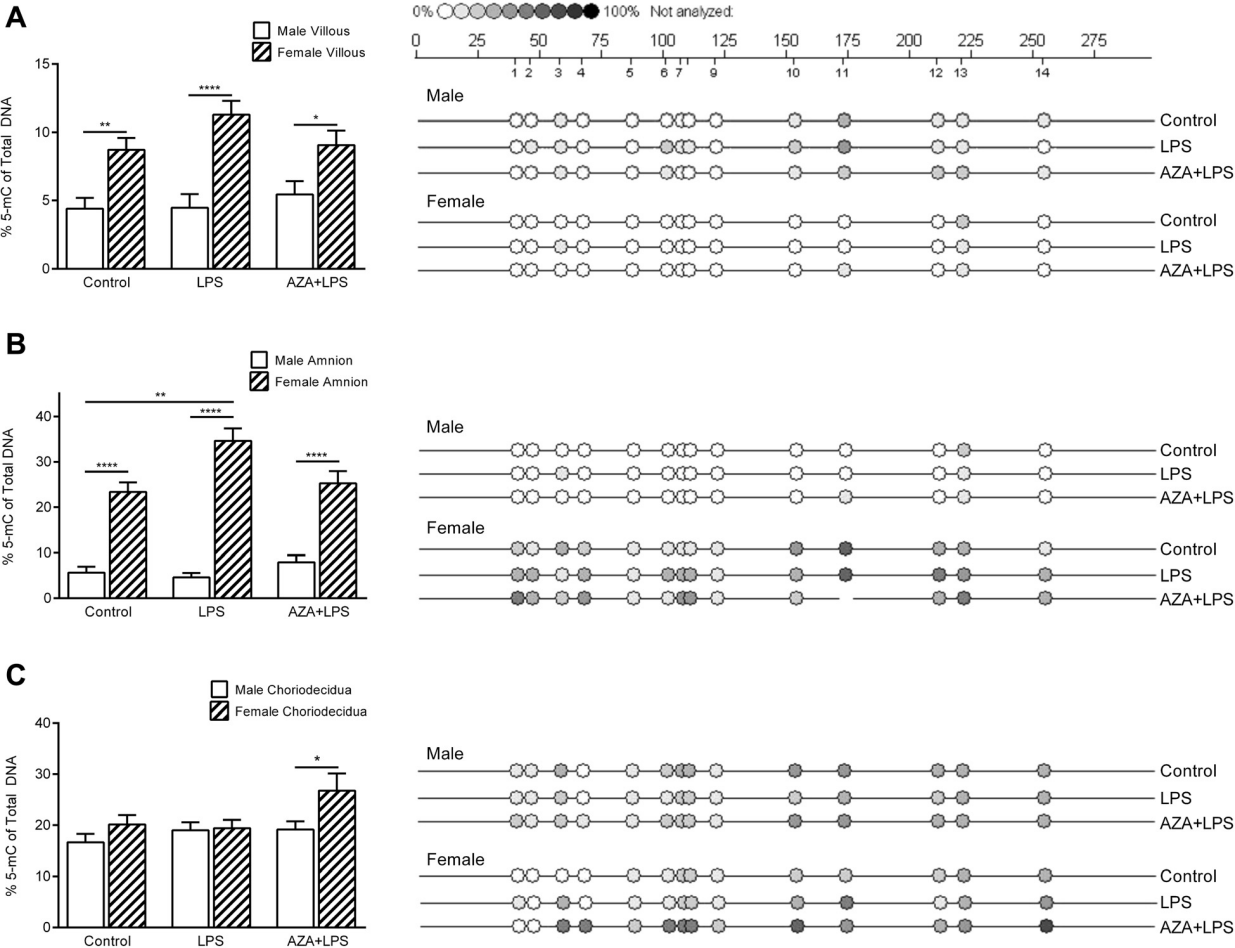
Percentage methylated CpGs in the TIMP-1 promoter in gestational tissues cultured in the presence of lipopolysaccharide (LPS, 5 μg/ml) with/without prior treatment with 5-aza-deoxycytidine (AZA, 5 μM) (*n* = 8). **a** Villous placenta; **b** Amnion and **c** Choriodecidua. Tissues from pregnancies with male fetuses (n = 5) are shown by white bars and those from pregnancies with female fetuses (n = 3) are shown by cross-hatched bars. For graphical representation, all CpGs within region A have been combined. Representative epigrams for both male and female tissues are shown alongside each graph. * P ≤ 0.05, ** P ≤ 0.01, **** P ≤ 0.001. Percentage methylation calculated from the ratio of mass signals between methylated and non-methylated DNA in each sample. Data are presented as mean %5-mC + SEM

Cultured villous placenta and amnion from pregnancies with male fetuses were hypomethylated compared to tissues from female pregnancies (on average: villous male 4.7 %, female 9.6 %; amnion male 5.35 %, female 29.5 %; Fig. 6a and b). Amnion from pregnancies with female fetuses cultured in the presence of LPS had significantly higher methylation compared to controls (34.6 % and 20.6 %, respectively; Fig. 6b). Methylation of choriodecidua from pregnancies with female fetuses was significantly higher following treatment with AZA and LPS compared to choriodecidua from pregnancies with male fetuses (26.8 % and 19.2 %, respectively; Fig. 6c).

## Discussion

The present study investigated the regulation of TIMP-1 by DNA methylation with or without infection in the human placenta and fetal membranes. We observed a labor effect on TIMP-1 transcription in villous placenta, and discovered sex-specific differential expression in amnion collected both prior to and post labor and delivery. There were subtle differences in methylation in all tissues in relation to labor status, which was discordant with the gene expression observed. The TIMP-1 promoter was generally hypomethylated, particularly in villous placenta, however sex specific methylation patterns were observed. Methylation in villous placenta and amnion from pregnancies with female fetuses was markedly higher than tissues from pregnancies with male fetuses, whereas the levels in choriodecidua were comparable. Treatment with demethylating agent AZA followed by incubation with LPS significantly increased TIMP-1 mRNA, and to a lesser extent protein, in cultured tissue explants. Villous placenta and amnion from pregnancies with male fetuses showed greater stimulation of TIMP-1 transcription following 48 hrs of treatment. This is the first study to show sex-specific expression patterns of TIMP-1 in term gestational tissues.

TIMP-1 is on the X chromosome and is consequently subject to X chromosome inactivation in females. The fact that higher methylation levels were observed in female tissues in this study was not surprising, as methylation of X-linked genes is enriched on the inactivated X chromosome [46, 47]. However, not all X-linked genes are completely silenced, and TIMP-1 is reported to display variable inactivation due to both changes in methylation and chromatin structure [48, 49]. With the exception of genes which do escape X inactivation, X-linked promoters should show limited methylation in males and partial methylation in females [50]. X-linked methylation explains the differences in methylation observed in tissues from pregnancies with male or female tissues collected prior to, and post labor and delivery, and the associated discordant gene expression seen in amnion samples. However, complete inactivation of one X-chromosome should result in a gene showing at least 50 % methylation, which was not the case in all tissues from pregnancies with female fetuses tested, thus suggesting variable inactivation.

A comparison of autosomal and X-lined genes showed that methylation of placental promoters is reduced only on the X chromosome, and is particularly evident in females resulting from an X- specific methylation decrease [51]. Due to the fact that X-linked placental genes are not usually over expressed, other epigenetic marks, such as chromatin modifications and non-coding RNA, are likely involved in the silencing of X-linked genes, including chromatin changes and non-coding RNA [52]. It is possible that other epigenetic marks are regulating TIMP-1 in the placenta.

The levels of methylation we observed in choriodecidua samples did not differ between tissues from pregnancies with male or female pregnancies. This could be explained in part by the contamination of adjacent maternal cells in the tissue sample. The chorion is closely associated to maternal decidua cells, which are firmly adhered to the chorion membrane. Due to tissue explant system used, it was not possible to separate the fetal cells from the maternal cells, thus no analysis could be carried out on the contribution of either cell’s contribution to the gene expression observed. It would therefore be an interesting avenue for future studies to investigate. Despite the presence of maternal cells in male tissue samples, as there is still one active allele in maternal tissues the X-linked methylation cannot alone account for the lessened response of TIMP-1 transcription and protein to treatments seen in cultured tissue explants from pregnancies with female fetuses when compared to tissues from pregnancies with male fetuses. However, the greater response observed in tissues from pregnancies with male fetuses could simply be due to them being more susceptible to the LPS treatment [53].

Tissue and sex-specific differential methylation was observed in both tissues collected at term prior to, and post labor and delivery, and in treated explants. Increased gene expression in the presence of greater methylation could be due to the mixture of silent methylated cells and expressing unmethylated cells in the tissues [54]. It is becoming increasing apparent that DNA methylation may not just function in gene silencing, increased methylation has been associated with enhanced gene expression for several genes including IL8, EGRF-2, HLA-DRA and GPH-α [55–59].

Aside from one time-point in choriodecidua explants, TIMP-1 transcription or protein was not changed with LPS treatment alone. This is in agreement with other studies which have also reported no change in TIMP-1 levels in the placenta and/or fetal membranes following LPS stimulation [17, 19, 60]. These studies did however report an associated increase in MMP-9, thereby altering the MMP:TIMP ratio and favouring a gelatonlytic state. Evidence of LPS associated stimulation of MMP-9 is conflicting; there are a number of reports of increased MMP-9 mRNA and activity in response to LPS stimulation, [5, 59, 61] whilst others report that fetal membranes are unresponsive to LPS, suggesting upstream mediators of MMPs such as prostaglandins and/or pro-inflammatory cytokines are required [61, 62].

AZA is a demethylating agent that is incorporated into the DNA as a cytidine analogue, preventing methylation by irreversibly binding DNMT1 [63]. The resulting loss of DNMT1 leads to passive hypomethylation of the genome over successive rounds of cell division, which may have effects on upstream regulators of TIMP-1. Treatment with AZA has previously been shown to induce TIMP-1 expression and protein production in cell culture models [39–43]. Treatment of tissue explants with AZA alone did not alter TIMP-1 expression or protein (data not shown). Our tissue culture model utilized tissue explants rather than cell lines, and in light of this, AZA may not have fully incorporated into the DNA, resulting in reduced action and less pronounced hypomethylation [64]. Although we did not measure proliferation in the tissue explants, glucose uptake by the tissue explants was measured in culture media and confirmed that tissues remained viable throughout the culture period (Table 1). AZA could be directly interacting with transcription factors [65] or re-organizing chromatin in such a way that regulatory regions within the promoter are exposed [66–68], thus facilitating binding of transcription factors which are increased in the presence of infection. We saw little variation in both TIMP-1 promoter-specific and global methylation (data not shown) in response to the explant treatments; therefore the increased TIMP-1 transcription observed in tissues pre-treated with AZA and subsequently cultured with LPS indicates that TIMP-1 activation appears to require an AZA-induced change in chromatin structure, such that DNA binding sites in the promoter region become accessible to transcriptional activators. The TIMP-1 promoter contains both ETS and AP-1 binding sites, which, once bound by effector proteins could potentiate enhanced TIMP-1 transcription [51]. Work on human hypoxanthine phosphoribosyltransferase (HPRT) and human-mouse phosphoglycerate kinase-1 (PK-1) genes suggest that X-linked genes are unlikely to be primarily silenced by DNA methylation via direct sequence-specific alterations in DNA-protein reactions; rather, methylation primarily affects chromatin structure [66–68].

TIMP-1 protein was not increased to the same extent as transcription following culture treatments. Discrepancies between TIMP-1 mRNA and protein have previously been reported [69]. and the amount of TIMP-1 translation is dependent upon the activation of a number of signalling pathways [70]. Furthermore, as TIMP-1 is secreted and has a short half-life, and as such the amount measured by western blotting in tissue explants only demonstrate the intracellular contribution of TIMP-1 production [71, 72].

The placenta itself is inherently variable, and significant intra-placental variation has been observed due to the vast range of cell types as well as normal variation in size, shape and weight [73]. There can also be considerable epigenetic variation within a placenta, so in addition to the effect of cell heterogeneity, there could also be random and localized effects of the uterine environment on the placental epigenome [73]. In light of this, every effort was taken to sample tissue widely across the placenta and fetal membranes.

## Conclusions

Intrauterine infection is the leading identifiable cause of preterm birth, closely followed by PROM, and despite the implementation of antibiotic treatments to reduce perinatal morbidity and mortality, the incidence of preterm birth is in fact increasing [74, 75]. The use of inhibitors of metalloproteinases, in particular TIMP-1, provides an attractive option as a potential therapeutic agent. The marked increase of TIMP-1 that we have seen in gestational tissues in response to LPS in the context of altered chromatin structure by AZA therefore suggests that a pharmacological agent that modifies chromatin structure could be used as a therapeutic agent in women with intrauterine infection and who are at a greater risk of premature rupture of the fetal membranes.
